# Brazilian version of the Personal Report of Communication Apprehension: Cross-cultural adaptation and psychometric evaluation among healthcare students

**DOI:** 10.1371/journal.pone.0246075

**Published:** 2021-02-04

**Authors:** Dyego Carlos Souza Anacleto de Araújo, Sylmara Nayara Pereira, Willian Melo dos Santos, Pedro Wlisses dos Santos Menezes, Kérilin Stancine dos Santos Rocha, Sabrina Cerqueira-Santos, André Faro, Alessandra Rezende Mesquita, Divaldo Pereira de Lyra

**Affiliations:** 1 Health Sciences Graduate Program, Federal University of Sergipe, São Cristóvão, Sergipe, Brazil; 2 Laboratory of Teaching and Research of Social Pharmacy, Federal University of Sergipe, São Cristóvão, Sergipe, Brazil; 3 Graduate Program in Pharmaceutical Sciences, Federal University of Sergipe, São Cristóvão, Sergipe, Brazil; 4 Graduate Program in Social Psychology, Health Psychology Laboratory (GEPPS/UFS) Federal University of Sergipe, São Cristóvão, Sergipe, Brazil; Guangzhou University, CHINA

## Abstract

**Introduction:**

Communication apprehension (CA) refers to an individual’s level of fear or anxiety toward either real or anticipated communication with another person or persons. The Personal Report of Communication Apprehension (PRCA-24) is the most widely used measure of CA, even among healthcare students.

**Objective:**

This study aimed to undertake a cross-cultural adaptation of this scale, translate it into Brazilian Portuguese, and examine its psychometric properties among healthcare students.

**Methods:**

The translation and cross-cultural adaptation procedures were undertaken with the objective of establishing compatibility between the original and translated scales. The content validity of the scale was established based on the feedback of a multidisciplinary expert committee. Its psychometric properties were evaluated using a convenience sample of 616 healthcare students. Its construct validity was examined using exploratory factor analysis (EFA) and confirmatory factor analysis (CFA). Its internal consistency was examined by computing Cronbach’s alpha and McDonald’s omega coefficients. Its criterion validity was examined against the Interpersonal Communication Competence Scale (ICCS).

**Results:**

The adapted scale demonstrated acceptable content validity. EFA showed that it was undergirded by one dimension, and this observation was confirmed by the results of CFA. The scale demonstrated excellent internal consistency. Its convergent validity was examined by conducting correlation analysis, and scores on the adapted PRCA-24 were negatively correlated with scores on the ICCS.

**Conclusion:**

The Brazilian version of the PRCA-24 has satisfactory psychometric properties and is, therefore, suitable for use with Brazilian healthcare students. It can be used to assess their communication needs for the purpose of designing tailored training programs.

## Introduction

Communication in healthcare has emerged as an important topic over the past few decades, and research studies have consistently found that communication skills are linked to patient satisfaction and better clinical outcomes [1,2]. Effective communication occurs when one possesses the knowledge and skills that are needed to communicate, is willing to communicate, and is afforded adequate opportunities to communicate. One’s willingness to communicate is influenced by certain factors such as shyness, self-perceived communication competence, and communication apprehension [3]. Past studies have used different approaches to impart communication skills to healthcare students. However, most of them have focused on skills training, and only few studies have examined the aforementioned psychological factors [4–8].

Communication apprehension is defined as “an individual’s fear or anxiety associated with either real or anticipated communication with another person or persons” [9]. It refers to how a person feels about communication, not how they communicate. In general, there are two types of communication apprehension: trait-like and state-like. Trait-like communication apprehension is defined as “a general pattern of low, moderate, or high orientation of anxiety/fear across communication contexts,” whereas state-like communication apprehension refers to “experiencing anxiety/fear in one situation but not others” [10–13]. Individuals who experience high levels of apprehension avoid communicating with others as much as possible in order to avoid experiencing the fear or anxiety that is associated with communication encounters [9]. Healthcare professionals who avoid communicating with their patients may not only be at a disadvantage professionally but also fail to provide extensive patient education.

This variable is commonly measured using the Personal Report of Communication Apprehension (PRCA-24). It is considered to be the most widely used measure of trait-like communication apprehension among both researchers and practitioners. The PRCA-24 covers four communication contexts: speaking in public, small groups, meetings, and dyads [14]. Past studies have demonstrated support for its strong internal consistency and construct, content, and criterion-related validity [15–18]. PRCA-24 has been widely used to measure communication apprehension among health profession students. However, this phenomenon had largely been confined to the pharmaceutical education enterprise in the United States (US) [19–27]. This observation underscores the need for further research among the students of other undergraduate courses and in other cultures.

With regard to Brazil, Araujo et al. have identified a few valid measures of communication among healthcare students, but none of them measure communication apprehension [28]. The measures of communication anxiety that are available in Brazilian Portuguese focus on public speaking [29–31]. Therefore, this study aimed to translate the PRCA-24 into Brazilian Portuguese and evaluate the psychometric properties of the cross-cultural adaptation among healthcare students.

## Methods

### Study design

Between June 2019 and March 2020, the cross-cultural adaptation and validation of the PRCA-24 were undertaken across three phases: 1) translation and cross-cultural adaptation, 2) the establishment of content validity, and 3) the evaluation of its psychometric properties.

### Phase 1: Translation and cross-cultural adaptation

The translation (into Brazilian Portuguese) and adaptation of the PRCA-24 were undertaken across several steps, in accordance with the procedure described by Borsa, Damásio, and Bandeira [32]. First, the PRCA-24 was independently translated from English into Brazilian Portuguese by two bilingual Brazilian translators who had lived in an English-speaking country. They had no prior exposure to the original English version of this instrument, and they were instructed to focus on the meaning rather than the literal expressions that were embedded within the text. The translated versions were evaluated by the authors, who subsequently undertook a synthesis of the instrument. A panel of Brazilians (i.e., one English teacher, three experts in validation studies, and three authors) compared the original and translated versions and assessed their semantic, idiomatic, cultural, and conceptual compatibility with the objective of creating a single version of the assessment.

The Brazilian version of the PRCA-24 was evaluated by a group of 10 healthcare students. A structured interview was conducted with these students to assess if they had experienced any difficulties in understanding the scale items. None of the items of the translated and adapted version of the assessment was perceived to be difficult to understand. The assessment was found to have face validity, and the contents were interpreted as intended. Back-translation was undertaken by two bilingual translators who were agnostic to the objective of our study. One of them was a native English speaker, and the other was a native Portuguese speaker. A communication apprehension expert from the US compared the back-translations to the original version and established the face validity of the assessment. Given that the two versions were found to be grammatically and semantically compatible, the instrument was considered to be ready for tests of content validity.

### Phase 2: Content validity

To examine content validity, independent experts were asked to evaluate the Brazilian version of the PRCA-24. The experts were selected based on an adaptation of Fehring’s scoring model. A minimum score of five points was required to warrant selection [33]. Eight experts examined the content validity of the assessment (i.e., two physicians, two psychologists, two speech therapists, two nurses, and one pharmacist). Most of them were women (n = 6, 75%) and had a PhD (n = 7, 87.5%). Their mean age was 46.75 ± 17.57 years, and their mean duration of professional experience in tool construction was 14.14 ± 13.84 years.

Before they evaluated the content validity of the assessment, the experts were provided with a written summary of the study’s aims and methods and a description of how they ought to assess content validity. An electronic form of the assessment, which remained active for 30 days, was emailed to the experts. They were required to record their scores on a 5-point Likert scale, which ranged from 1 to 5. They were asked to rate each item on the following criteria: language clarity (i.e., the language is clear, understandable, and appropriate for the target population), practical pertinence (i.e., “the item assesses a concept that is of interest to the target population”), and theoretical relevance (i.e., the relevance between the items and the underlying theory). The experts were also invited to provide additional comments about each scale item.

The content validity coefficient was computed to quantify content validity. The cutoff score that was used to indicate a satisfactory level of language clarity, practical pertinence, and theoretical relevance was ≥ .80 (for each item and the entire scale) [34]. On all the three criteria, the adapted PRCA-24 obtained coefficients that were > .80. (range = .85–1.0). Since all the items were found to be clear and relevant to Brazilian healthcare students, all the items were retained. The experts also provided formative feedback in their written comments. Their feedback was discussed among the authors and subsequently incorporated into the final draft of the Brazilian version of the PRCA-24. The main suggestions pertained to the randomization of the items in an order that differs from that of the original scale and the inversion of the rating scale.

### Phase 3: Evaluation of psychometric properties

#### Participants

A convenience sample of students who were enrolled in the undergraduate healthcare courses that were offered by the Federal University of Sergipe (Brazil) responded to the Brazilian version of the PRCA-24. Those who were older than 18 years and were dental, pharmaceutical, medical, or nursing students at the Federal University of Sergipe in São Cristóvão, Sergipe, Brazil, were eligible for inclusion. They were invited to voluntarily respond to the instrument during class hours between December 2019 and March 2020. In psychometric studies, a minimum of 25 respondents per item is required. Accordingly, the minimum required sample size was found to be 600 [35]. Although participation was voluntary, none of the students declined the invitation to respond to the assessment. The participants were 616 students (women: n = 373, 60.6%) aged 18–49 years (M = 21.76, SD = 3.61). Further, 37.8% (n = 233), 32.6% (n = 201), 20.1% (n = 124), and 9.4% (n = 58) of them were pharmaceutical, medical, nursing, and dental students, respectively.

#### Instruments

*Brazilian version of the PRCA-24*. This 24-item scale assesses trait-like communication apprehension across four communication contexts: dyadic, meeting, public, and small groups. Respondents are required to indicate their level of agreement with each statement on a 5-point Likert-type scale. Total scores can range from 24 to 120. Lower and higher scores are indicative of lower and higher levels of communication apprehension, respectively [36] (S1 Appendix).

*Interpersonal Communication Competence Scale (ICCS)*. [37] The Brazilian version of the ICCS has been developed and validated by Puggina and Silva [38]. This 17-item scale assesses subjectively perceived competence in relation to interpersonal communication. Respondents are required to indicate their level of agreement with each item on a 5-point Likert-type scale. Higher scores are indicative of positive subjective perceptions of one’s interpersonal communication skills.”

#### Data analysis

*Exploratory factor analysis (EFA)*. Since this was the first study to examine the psychometric properties of the (Brazilian version of the) PRCA-24 using a Brazilian sample, its construct validity was first tested using an exploratory methodology to identify the underlying theoretical dimensions. The 616 participants were randomly divided into two groups using an algorithm that is available in IBM SPSS® (version 22). The data collected from one group were used to conduct EFA (n = 240), and the data collected from the other group were used to conduct confirmatory factor analysis (CFA; n = 376). EFA was conducted using Factor (version 10.9.02) [39] the weighted least squares mean and variance adjusted extraction method, and robust promin rotation [40,41]. The robustness of the test was determined using bootstrapping (samples = 5,000). The factorability of the instrument was examined using the following criteria: the Kaiser-Meyer-Olkin (KMO) measure of sampling adequacy > .60 and significant results yielded by Bartlett’s test of sphericity (p < .05) [42,43].

The number of dimensions that had to be retained was determined by computing parallel analysis with permutations of sample values [44]. This analysis is considered to be one of the most robust and accurate means of testing dimensionality [45–48]. If the results of EFA indicate that one dimension should be extracted, the results should be accepted if the following criteria are met: unidimensional congruence (UNICO > .95), explained common variance (ECV > .85), and the mean of item residual absolute loadings (MIREAL < .30) [49]. The quality and effectiveness of the factor solution were evaluated based on the factor determinacy index (> .90) and expected percentage of true differences (> 90%) [49]. The distribution of the residuals was evaluated by inspecting the weighted root mean square residual; values that are < 1.0 are considered to be indicative of a good fit [50].

The internal consistency of the adapted PRCA-24 was examined by computing Cronbach’s alpha and McDonald’s omega coefficients [51,52]. Values that are ≥ .70, ≥ .80, and ≥ .90 are indicative of acceptable, good, and excellent internal consistency [53]. The use of two indicators increases the reliability of the interpretation, because inconsistencies in reliability can result from sole reliance on Cronbach’s alphas [54–56].

*Confirmatory factor analysis (CFA)*. CFA was conducted using JASP (version 0.12.1) and robust diagonally weighted least squares. Model fit was examined by inspecting the following indices: the ratio of the chi-squared statistic to the degrees of freedom (χ^2^/df) < 3, comparative fit index (CFI) ≥ .95 [57], Tucker-Lewis index (TLI) ≥ .95 [58], goodness of fit index ≥ .95, non-normed fit index (NNFI) ≥ .95, root mean square error of approximation (RMSEA) ≤ .08, p-close > .05 [59] and root mean square of residuals ≤ 0.8.

*Normalization*. The test scores were normalized by computing T-scores using the following formula: T-score = (Z-score × 10) + 50. The Z-scores were computed by dividing the difference between the raw score and the sample mean by the sample standard deviation (35). Independent-samples t-test was used to examine gender differences in scores on the adapted PRCA-24 (p ≤ .05). We calculated the percentile ranks (5% intervals) for the raw scores and T-scores.

*Convergent validity*. Pearson’s correlation analysis was conducted to examine the convergent validity of the adapted PRCA-24 against the ICCS. A moderate negative correlation was expected to emerge between the adapted PRCA-24 and ICCS scores.

### Ethical considerations

This study adhered to the ethical principles outlined in Resolution 466/12 of the National Board of Health and was approved by the Ethics Committee of the Federal University of Sergipe, Aracaju-Sergipe (Protocol n° 3.588.162). Written and oral information about the purpose of the study was provided to each participant. Participation in the study was voluntary and anonymous. Written informed consent was obtained from the participants prior to data collection.

## Results

### EFA

The significant results yielded by Bartlett’s test of sphericity (3626.2, df = 276, *p* < 0.001) and the emergent KMO statistic (.95) supported the appropriateness of using factor analysis (Table 1). Parallel analysis showed that the scale was undergirded by one dimension, which explained 56.3% of the variance (see Table 2 for the factor loadings). This indicated that the model had high explanatory power. The unidimensionality of the scale was supported by the following results: UNICO = .95, ECV = .90, and MIREAL = .20. All these results further confirmed that the items were undergirded by a unidimensional variable. Both the Cronbach’s α and McDonald’s ω coefficients were .96, and this value is greater than the minimum acceptable threshold of .70 (Table 1).

**Table 1:** Summary of the model characteristics.

**Exploratory**	Adequacy of correlation matrix	Bartlett	3626.2 (df = 276)^a^
		Kaiser-Meyer-Olkin (KMO)	.95
	Dimensions (Parallel analysis)		1
	Explained Variance		56.3%
	Quality and effectiveness of factor score estimates	Factor Determinacy Index (FDI)	.98
		Expected percentage of true differences (EPDT)	96.7%
	Distribution of residuals	Weighted Root Mean Square Residual (WRSR)	.05
**Dimensionality**	Unidimensional Congruence (UNICO)		.95
	Explained Common Variance (ECV)		.90
	Mean of item residual absolute loading (MIREAL)		.20
**Confirmatory**	Chi-square ratio for degree of freedom (X2/df)		1.48
	Non-Normed Fit Index (NNFI)		.99
	Comparative Fit Index (CFI)		.99
	Tucker-Lewis Index (TLI)		.99
	Goodness of Fit Index (GFI)		.98
	Root Mean Square Error of Approximation (RMSEA)-[C.I. 90%]		.036 [.028-.043]^b^
	Standardized Root Mean squared Residual (SRMR)		.06
**Reliability**	Standardized Cronbach’s Alpha		.96
	McDonald’s Omega		.96

^a^ *p* < .0001

^b^ *p* = .99.

**Table 2:** EFA results: Items and factor loadings for the one-dimension structure of Personal Report of Communication Apprehension (PRCA-24).

	Factor load
Item 1	-.59
Item 2	.75
Item 3	-.60
Item 4	.73
Item 5	-.68
Item 6	.36
Item 7	-.78
Item 8	.79
Item 9	-.48
Item 10	.83
Item 11	-.82
Item 12	.72
Item 13	-.74
Item 14	.65
Item 15	-.72
Item 16	.70
Item 17	-.78
Item 18	.82
Item 19	-.78
Item 20	.73
Item 21	-.78
Item 22	.64
Item 23	-.79
Item 24	.85

### CFA

The model fit indices that were yielded by CFA supported the hypothesized unidimensional structure of the scale and indicated that it did not require any modification (χ^2^/df = 1.48, CFI = .99, TLI = .98, RMSEA = .036, 90% CI = .028–.043). The NNFI indicated that the 1-factor model demonstrated a 99% improvement in fit when compared to the null model, and this result was consistent with the other indices (Table 1).

### Communication apprehension: Total scores

After establishing the appropriateness of the unidimensional model for the present sample, an algorithm was created to calculate the total scores on the adapted PRCA-24. Responses to items 2, 4, 6, 8, 10, 12, 14, 16, 18, 20, 22, and 24 were recoded, and all the individual item scores were summed.

### Normalization

Total scores on the adapted PRCA-24 were calculated for all the students and normalized (Table 3). The T-score has a standardized mean of 50 and standard deviation of 10. This permits the standardization of raw scores along a scale that ranges from 0 to 100. The 25th and 75th percentiles were computed to identify the participants who reported extremely low and high levels of communication apprehension, respectively. Based on the 25th, 50th, and 75th percentiles, the participants were classified into three groups: low (total: ≤ 59), moderate (total: 60–84), and high (total: ≥ 85) scores.

**Table 3:** Norm table for the Personal Report of Communication Apprehension.

Percentile rank	Raw score	T Score
5	40	32
10	48	37
15	52	39
20	55	41
25	59	43
30	62	45
35	65	46
40	67	47
45	70	49
50	73	51
55	75	52
60	77	53
65	80	55
70	82	56
75	85	57
80	88	59
85	90	60
90	95	63
95	100	66

### Convergent validity

Correlation analysis was conducted to examine the convergent validity of the adapted PRCA-24 among healthcare students. Scores on the adapted PRCA-24 were negatively correlated with scores on the ICCS (*r* = -.56, *p* < .001).

## Discussion

In this study, the PRCA-24 was adapted to the Brazilian context and translated into Brazilian Portuguese. The translated version was refined based on the feedback of a panel of experts. Its psychometric properties were examined using rigorous and advanced data analytic techniques. The Brazilian version of the PRCA-24 was found to be reliable and valid among healthcare students. The assessment demonstrated acceptable content, construct, and criterion validity and reliability. It was adapted to the local culture, and it was found to be easy to understand, clear, and relevant. The adapted PRCA-24 can be used for educational and research purposes. Further, it can be used to help health profession students better understand their communication apprehension with the objective of reinforcing their strengths and identifying areas that require more attention. It can also be used to assess healthcare students’ communication needs for the purpose of designing tailored training programs.

During the content validation process, some changes were made to the instrument’s structure. In the original version, items pertaining to the same context are listed sequentially (group: items 1–6; meeting: items 7–12; dyadic: items 13–18; public speaking: items 19–24). In the Brazilian version, the items are arranged in an alternate order based on the context and meaning of the statement (i.e., positive or negative). In addition to avoiding redundancy, the sequential presentation of positively and negatively worded items pertaining to the same context makes inconsistencies in responses more readily apparent. Moreover, the Likert scale was modified in such a manner that it ranged from “strongly disagree” (1) to “strongly agree” (5). This is the rating scale that is most frequently used in Brazil. These changes rendered the scale contents clearer and relevant to the local culture.

EFA and CFA showed that the Brazilian version of the PRCA-24 was unidimensional in nature. This interpretation was supported by the emergent UNICO, ECV, and MIREAL values, excellent model fit indices, and high explained variance values. This indicates that, regardless of the communication situation, the scale measures a single construct, which is communication apprehension. By subjecting data collected from more than 10,000 participants to factor analysis, McCroskey and Beatty [60] demonstrated support for the unidimensionality of this scale; other studies have also demonstrated support for the unidimensionality of this scale [61–64]. It is noteworthy that different structural models, ranging from a one-dimensional model to a four-dimensional model, have been documented in the literature [16,65,66]. The Brazilian version should be used as a global measure and we recommended that the four contexts are not evaluated independently.

Percentile ranks were computed for the total sample. An important feature of the normative tables is that they permit comparisons of individual scores (i.e., as percentile ranks or percentiles) against reference groups, comparisons between populations, and the establishment of parameters to interpret variability in this phenomenon.

The adapted PRCA-24 demonstrated excellent reliability, which was assessed by computing Cronbach’s α and McDonald’s ω coefficients. The limitations of Cronbach’s α have been described in the literature [67,68]. In this regard, the both indices supported the reliability of the Brazilian version of the PRCA-24. Similar scores were obtained in studies conducted using the original scale and US samples; the alpha coefficients ranged from .80 to .95 [16,17,69–73]. Similar scores were also obtained in studies conducted in other countries [65,74–76] A recent multinational study conducted by Croucher et al. (2019) also found that the PRCA-24 demonstrated strong reliability across different languages (i.e., French, German, Spanish, German, Kurdish, English, Russian) and national cultures (i.e., France, Germany, India, Iran, Kyrgyzstan, Rwanda, Spain, United Kingdom) [77]. These findings suggest that the PRCA-24 is highly reliable among both US and non-US samples.

The significant negative correlation that emerged between scores on the adapted PRCA-24 and ICCS is consistent with past findings. This finding suggests that communication apprehension is strongly and inversely related to self-perceived communication competence [69,78]. A meta‐analytic study reported that a negative relationship between communication apprehension and communication skills has consistently been found and the findings suggest that, as a person becomes more apprehensive, both the quantity and quality of his or her communication behaviors decline [79]. Since an inverse correlation was expected, these results indicate the convergent validity of the instrument. It is important to note that only the correlation analysis between PRCA-24 and ICCS is not sufficient, and future studies should evaluate the relationship with other scales. This study has some limitations, which need to be acknowledged. First, the study was conducted at a single center using a convenience sample of healthcare students from Sergipe in Brazil. This limits the generalizability of the results. Second, the evaluation was confined to one approach, and stability could not be tested because this study adopted a cross-sectional design. Third, the instrument’s ability to predict specific outcomes that are related to the phenomenon (e.g., generalized anxiety disorder or social anxiety) was not evaluated. Fourth, the invariance test was not performed because the small sample size and convenience sampling.

## Conclusion

The Brazilian version of the PRCA-24 was found to be an adequate cross-cultural adaptation of the original scale. Its psychometric properties were satisfactory, and it proved to be a simple and reliable measure of communication apprehension among healthcare students who speak Brazilian Portuguese. The adapted PRCA-24 can be used for educational and research purposes among healthcare students. Future studies should consider the limitations of this study, investigate the properties of the scale among healthcare students from different regions, analyze the stability of the scale using longitudinal data, and establish morphological, intercept and factor load equivalence.

## Supporting information

S1 Appendix Portuguese version of the Personal Report of Communication Apprehension (RePAC-24). (DOCX)

S2 Appendix Original version of the Personal Report of Communication Apprehension (PRCA-24). (PDF)
