# Correction: Brazilian version of the Personal Report of Communication Apprehension: Cross-cultural adaptation and psychometric evaluation among healthcare students

**DOI:** 10.1371/journal.pone.0258392

**Published:** 2021-10-04

**Authors:** Dyego Carlos Souza Anacleto de Araújo, Sylmara Nayara Pereira dos Santos, Willian Melo dos Santos, Pedro Wlisses dos Santos Menezes, Kérilin Stancine Santos Rocha, Sabrina Cerqueira-Santos, André Faro, Alessandra Rezende Mesquita, Divaldo Pereira de Lyra

The second author’s name is spelled incorrectly. The correct name is: Sylmara Nayara Pereira dos Santos. Additionally, the fifth author’s name is spelled incorrectly. The correct name is: Kérilin Stancine Santos Rocha. Finally, In the PDF version of the article, the ninth author’s name appears incorrectly. The correct name is: Divaldo Pereira de Lyra Jr.

The correct citation is: de Araújo DCSA, dos Santos SNP, dos Santos WM, dos Santos Menezes PW, Rocha KSS, Cerqueira-Santos S, et al. (2021) Brazilian version of the Personal Report of Communication Apprehension: Cross-cultural adaptation and psychometric evaluation among healthcare students. PLoS ONE 16(2): e0246075. https://doi.org/10.1371/journal.pone.0246075
