# Supplementary material for: Brazilian version of the Personal Report of Communication Apprehension: Cross-cultural adaptation and psychometric evaluation among healthcare students
Source: PLoS One. 2021 Feb 4;16(2):e0246075. doi: 10.1371/journal.pone.0246075 (PMC7861414; doi:10.1371/journal.pone.0246075)
Supplement: S1 Appendix — (DOCX) [file pone.0246075.s001.docx]

**INSTRUÇÕES:** Este instrumento é composto por 24 afirmações relacionadas a sentimentos experimentados por você ao se **comunicar presencialmente** com outras pessoas.

| *Indique seu grau de concordância com cada uma das afirmações. Marque apenas uma alternativa para cada item e certifique-se que todos foram respondidos.* | | | | |
| --- | --- | --- | --- | --- |
| Discordo fortemente | **Discordo** | **Não concordo, nem discordo** | **Concordo** | **Concordo fortemente** |
| 1 | **2** | **3** | **4** | **5** |

| Não gosto de participar de discussões em grupo. | 1 | 2 | 3 | 4 | 5 |
| --- | --- | --- | --- | --- | --- |
| Eu fico calmo e relaxado ao falar em público. | 1 | 2 | 3 | 4 | 5 |
| Participar de uma conversa com pessoas que acabei de conhecer me deixa muito nervoso. | 1 | 2 | 3 | 4 | 5 |
| Normalmente, eu me sinto confortável quando preciso participar de uma reunião. | 1 | 2 | 3 | 4 | 5 |
| Meus pensamentos ficam confusos e fora de ordem quando falo em público. | 1 | 2 | 3 | 4 | 5 |
| Quando estou participando de uma conversa, não tenho medo de falar o que penso. | 1 | 2 | 3 | 4 | 5 |
| Geralmente, eu fico nervoso quando tenho que participar de uma reunião. | 1 | 2 | 3 | 4 | 5 |
| Eu fico calmo e relaxado quando participo de discussões em grupo. | 1 | 2 | 3 | 4 | 5 |
| Tenho receio de falar o que penso quando estou participando de uma conversa. | 1 | 2 | 3 | 4 | 5 |
| Eu me sinto confiante quando tenho que falar em público. | 1 | 2 | 3 | 4 | 5 |
| Participar de discussões em grupo me deixa tenso e nervoso. | 1 | 2 | 3 | 4 | 5 |
| Eu fico muito tranquilo quando respondo perguntas em uma reunião. | 1 | 2 | 3 | 4 | 5 |
| Falar em reuniões geralmente me deixa desconfortável. | 1 | 2 | 3 | 4 | 5 |
| Na maioria das vezes, eu fico bem tranquilo e relaxado em conversas. | 1 | 2 | 3 | 4 | 5 |
| Sinto meu corpo tenso e rígido quando preciso falar em público. | 1 | 2 | 3 | 4 | 5 |
| Eu gosto de fazer parte de discussões em grupo. | 1 | 2 | 3 | 4 | 5 |
| Geralmente, participar de conversas me deixa muito tenso e nervoso. | 1 | 2 | 3 | 4 | 5 |
| Eu não tenho medo de falar em público. | 1 | 2 | 3 | 4 | 5 |
| Eu tenho receio de me expressar durante uma reunião. | 1 | 2 | 3 | 4 | 5 |
| Eu fico muito tranquilo e relaxado quando me pedem para expressar minha opinião em uma reunião. | 1 | 2 | 3 | 4 | 5 |
| Participar de uma discussão em grupo com pessoas que não conheço me deixa tenso e nervoso. | 1 | 2 | 3 | 4 | 5 |
| Eu fico muito tranquilo ao conversar com pessoas que acabei de conhecer. | 1 | 2 | 3 | 4 | 5 |
| Ao falar em público, fico tão nervoso que esqueço o que sei. | 1 | 2 | 3 | 4 | 5 |
| Geralmente, eu me sinto confortável ao participar de discussões em grupo. | 1 | 2 | 3 | 4 | 5 |
