# Supplementary material for: Brazilian version of the Personal Report of Communication Apprehension: Cross-cultural adaptation and psychometric evaluation among healthcare students
Source: PLoS One. 2021 Feb 4;16(2):e0246075. doi: 10.1371/journal.pone.0246075 (PMC7861414; doi:10.1371/journal.pone.0246075)
Supplement: S2 Appendix — (PDF) [file pone.0246075.s002.pdf]

Personal Report of Communication Apprehension (PRCA-24)

DIRECTIONS: This instrument is composed of twenty-four statements concerning feelings about communicating with other people. Please indicate the degree to which each statement applies to you by marking whether you **strongly agree (1-SA), agree (2-A), undecided (3-U), disagree (4-D), or strongly disagree (5-SD).**

Work quickly; record your first impression.

Question	Response				
1. I dislike participating in group discussions.	1 - SA	2 - A	3 - U	4 - D	5 - SD
2. Generally, I am comfortable while participating in group discussions.	1 - SA	2 - A	3 - U	4 - D	5 – SD
3. I am tense and nervous while participating in group discussions.	1 - SA	2 - A	3 - U	4 - D	5 - SD
4. I like to get involved in group discussions.	1 - SA	2 - A	3 - U	4 - D	5 - SD
5. Engaging in a group discussion with new people makes me tense and nervous.	1 - SA	2 - A	3 - U	4 - D	5 - SD
6. I am calm and relaxed while participating in group discussions.	1 - SA	2 - A	3 - U	4 - D	5 - SD
7. Generally, I am nervous when I have to participate in a meeting.	1 - SA	2 - A	3 - U	4 - D	5 - SD
8. Usually I am calm and relaxed while participating in meetings.	1 - SA	2 - A	3 - U	4 - D	5 - SD
9. I am very calm and relaxed when I am called upon to express an opinion at a meeting.	1 - SA	2 - A	3 - U	4 - D	5 - SD
10. I am afraid to express myself at meetings.	1 - SA	2 - A	3 - U	4 - D	5 - SD
11. Communicating at meetings usually makes me uncomfortable.	1 - SA	2 - A	3 - U	4 - D	5 - SD
12. I am very relaxed when answering questions at a meeting.	1 - SA	2 - A	3 - U	4 - D	5 - SD
13. While participating in a conversation with a new acquaintance, I feel very nervous.	1 - SA	2 - A	3 - U	4 - D	5 - SD
14. I have no fear of speaking up in conversations.	1 - SA	2 - A	3 - U	4 - D	5 - SD
15. Ordinarily I am very tense and nervous in conversations.	1 - SA	2 - A	3 - U	4 - D	5 - SD
16. Ordinarily I am very calm and relaxed in conversations.	1 - SA	2 - A	3 - U	4 - D	5 - SD
17. While conversing with a new acquaintance, I feel very relaxed.	1 - SA	2 - A	3 - U	4 - D	5 - SD
18. I'm afraid to speak up in conversations.	1 - SA	2 - A	3 - U	4 - D	5 - SD
19. I have no fear of giving a speech.	1 - SA	2 - A	3 - U	4 - D	5 - SD
20. Certain parts of my body feel very tense and rigid while giving a speech.	1 - SA	2 - A	3 - U	4 - D	5 - SD
21. I feel relaxed while giving a speech.	1 - SA	2 - A	3 - U	4 - D	5 - SD
22. My thoughts become confused and jumbled when I am giving a speech.	1 - SA	2 - A	3 - U	4 - D	5 - SD
23. I face the prospect of giving a speech with confidence.	1 - SA	2 - A	3 - U	4 - D	5 - SD
24. While giving a speech, I get so nervous I forget facts I really know.	1 - SA	2 - A	3 - U	4 - D	5 - SD

Personal Report of Communication Apprehension Scoring

SCORING: Compute subscores for four communication contexts–group discussions, meetings, interpersonal conversations, and public speaking– and an overall communication apprehension (CA) score. Strongly agree=1 point, agree=2 points, undecided=3 points, etc.

Sub scores	Scoring Formula
Group discussion	18+scores for items 2, 4, and 6;<br>– scores for items 1, 3, and 5
Meetings	18+scores for items 8, 9, and 12;<br>– scores for items 7, 10, and 11
Interpersonal conversations	18+scores for items 14, 16, and 17;<br>– scores for items 13, 15, and 18
Public speaking	18+scores for items 19, 21, and 23;<br>– scores for items 20, 22, and 24

Scores on the four contexts (groups, meetings, interpersonal conversations, and public speaking) can range from a low of 6 to a high of 30. Any score above 18 indicates some degree of apprehension.

To determine your overall CA score, add together all four sub scores.

Your score should range between 24 and 120. If your score is below 24 or above 120, you have made a mistake in computing the score.

Scores between **83 and 120** indicate a high level of communication apprehension.

Scores between **55 and 83** indicate a moderate level of communication apprehension.

Scores between **24 and 55** indicate a low level of communication apprehension.
